# Costs of potentially inappropriate medication use in residential aged care facilities

**DOI:** 10.1186/s12877-018-0704-8

**Published:** 2018-01-11

**Authors:** S. L. Harrison, L. Kouladjian O’Donnell, R. Milte, S. M. Dyer, E. S. Gnanamanickam, C. Bradley, E. Liu, S. N. Hilmer, M. Crotty

**Affiliations:** 1 Department of Rehabilitation, Aged and Extended Care, Faculty of Medicine, Nursing and Health Sciences, School of Health Sciences, Flinders University, Level 4, Rehabilitation Building, Flinders Medical Centre, Flinders Drive, Bedford park, SA 5042 Australia; 2 NHMRC Cognitive Decline Partnership Centre, University of Sydney, Sydney, NSW Australia; 3 Kolling Institute of Medical Research, University of Sydney and Royal North Shore Hospital, St Leonards, NSW 2065 Australia; 4 Institute for Choice, University of South Australia, GPO Box 2471, Adelaide, SA 5001 Australia; 5 Infection & Immunity – Aboriginal Health, SAHMRI, PO Box 11060, Adelaide, SA 5001 Australia; 6 Mary MacKillop Institute for Health Research, Australian Catholic University, 215 Spring Street, Melbourne, VIC 3000 Australia

**Keywords:** Beers criteria, Potentially inappropriate medications, Older adults, Cognitive impairment, Dementia

## Abstract

**Background:**

The potential harms of some medications may outweigh their potential benefits (inappropriate medication use). Despite recommendations to avoid the use of potentially inappropriate medications (PIMs) in older adults, the prevalence of PIM use is high in different settings including residential aged care. However, it remains unclear what the costs of these medications are in this setting. The main objective of this study was to determine the costs of PIMs in older adults living in residential care. A secondary objective was to examine if there was a difference in costs of PIMs in a home-like model of residential care compared to an Australian standard model of care.

**Methods:**

Participants included 541 participants from the Investigation Services Provided in the Residential Environment for Dementia (INSPIRED) Study. The INSPIRED study is a cross-sectional study of 17 residential aged care facilities in Australia. 12 month medication costs were determined for the participants and PIMs were identified using the 2015 updated Beers Criteria for older adults.

**Results:**

Of all of the medications dispensed in 1 year, 15.9% were PIMs and 81.4% of the participants had been exposed to a PIM. Log-linear models showed exposure to a PIM was associated with higher total medication costs (Adjusted β = 0.307, 95% CI 0.235 to 0.379, *p* < 0.001). The mean proportion (±SD) of medication costs that were spent on PIMs in 1 year was 17.5% (±17.8) (AUD$410.89 ± 479.45 per participant exposed to a PIM). The largest PIM costs arose from proton-pump inhibitors (34.4%), antipsychotics (21.0%) and benzodiazepines (18.7%). The odds of incurring costs from PIMs were 52% lower for those residing in a home-like model of care compared to a standard model of care.

**Conclusions:**

The use of PIMs for older adults in residential care facilities is high and these medications represent a substantial cost which has the potential to be lowered. Further research should investigate whether medication reviews in this population could lead to potential cost savings and improvement in clinical outcomes. Adopting a home-like model of residential care may be associated with reduced prevalence and costs of PIMs.

**Electronic supplementary material:**

The online version of this article (10.1186/s12877-018-0704-8) contains supplementary material, which is available to authorized users.

## Background

Polypharmacy, i.e. the use of five or more medications, is high in residential aged care facilities [1]. Furthermore, the potential harms of some medications may outweigh their potential benefits (inappropriate medication use). Inappropriate polypharmacy is not only associated with an increased risk of adverse drug reactions, but also an increased risk of disability, unplanned hospitalizations and mortality [2].

In 2015 the Beers Criteria for potentially inappropriate medication (PIM) use in older adults were updated by the American Geriatrics Society [3]. The Beers Criteria provide a comprehensive list of PIMs; the majority of the medications included in the Beers Criteria are strongly recommended to be avoided in older adults, and the evidence to support these recommendations was largely rated as moderate or high quality [3]. PIMs, according to the Beers Criteria, have been associated with increased risks of hospitalization and mortality in older people in residential care [4]. In addition to their potential adverse health effects and negative effect on quality of life, the use of PIMs may also lead to higher health care costs [5, 6].

An additional Beers Criteria list specific for older adults with dementia or cognitive impairment is also included in the 2015 update. The evidence to support the development of this list was rated as moderate quality by the authors of the Beers Criteria. The authors strongly recommend avoiding medications within this list in older adults with dementia or cognitive impairment because these medications may cause adverse central nervous system effects, and because of the association of antipsychotics with a greater risk of stroke and mortality in people with dementia [3].

Despite recommendations to avoid the use of PIMs in older adults, the prevalence of PIM use has been shown to be high in a number of different settings including residential aged care. Approximately one half of aged care residents have been previously shown to be exposed to PIMs [7]. However, it remains unclear what the costs of these medications are in this setting.

It has not previously been shown if different models of residential care can affect the use of PIMs. Models of residential care for older adults that aim to provide small group home-like environments and encourage independence for the residents have been of increasing interest [8, 9]. The World Health Organization stated that these models ‘hold promise’ for the residents, family members and staff [9]. However, the effectiveness of these home-like residential care models needs to be further examined [10]. Living in home-like residential care models may improve quality of life for residents [8], whether this is reflected in exposure to PIMs has not previously been investigated.

The objectives of this study were to determine exposure to and cost of PIMs according to the 2015 updated Beers Criteria for all older adults living in residential care and the additional criteria for people living with cognitive impairment and dementia. Further, we examined if there was a difference in the amount spent on these medications in a home-like residential care model compared to a standard model of care.

## Methods

### Study participants

The Investigating Services Provided in the Residential Environment for Dementia (INSPIRED) study is a cross-sectional observational study of residential aged care facilities in Australia and was designed to include participants with cognitive impairment and dementia. The study received ethics approval from the Flinders Social and Behavioural Research Ethics Committee. The study aimed to include facilities from areas representing different socioeconomic backgrounds, geographic locations (e.g. rural vs metropolitan locations) and different states of Australia. In total, 17 facilities in Australia (including South Australia, New South Wales, Western Australia, and Queensland) participated in the study. Participants self-consented to be involved in the study or, where more severe cognitive impairment was present, informed consent and information was provided by a proxy, usually a close family member (76% of the participants). To be eligible for participation in the study the participants needed to: 1) have been a permanent resident in the facility for 12 months or more, 2) have not been in immediate palliative care, 3) have no complex medical or family issues which would impede their participation and 4) have a family member available and willing to participate on their behalf if a proxy was required.

In total, 1323 potential participants were assessed for eligibility and 901 were eligible to participate; of these 60% (*n* = 541) consented. Data collection was completed between January 2015 and February 2016.

### Medication use

Medication use was primarily based on dispensing records obtained from the appropriate pharmacy. Data for dispensing of the medication in the 365 days prior to the study start date for the facility was collected (hereafter referred to as 12 month medication records). Of the study participants, 3.5% (*n* = 19) did not have pharmacy records available, and reviews of their medication charts were undertaken instead. For two of the facilities (both standard model facilities) the pharmacy records only reported a single instance for the initial dispensing date of the medication, rather than all dates of provision of the medication, hence, Pharmaceutical Benefits Scheme (PBS) records were used to determine medication use and costs. In Australia, under the PBS, the government subsidises costs of many prescription medications for Australian residents. Accordingly, the majority of dispensed medications will be found in PBS records.

### Potentially inappropriate medications

PIMs were identified using the 2015 updated Beers Criteria [3]. Only the medications which are listed as PIMs for all older adults (i.e. not those which are dependent on diagnosis) were considered for analysis of utilisation in all participants. Participants were deemed to have been exposed to a PIM if they had been prescribed at least one medication included in the Beers Criteria in the previous 12 months. Furthermore, we also completed a subgroup analysis to examine the Beers Criteria specific for people with cognitive impairment and dementia in addition to the Beers Criteria for all older adults. Only participants with a Psychogeriatric Assessment Scale-Cognitive Impairment Scale (PAS-Cog) score of more than 4 and/or a formal diagnosis of dementia were included in this secondary analysis. As the Beers Criteria were developed for the USA, some medications were added to the PIMs lists by research pharmacists to allow for the Australian register of pharmaceutical products; these medications were in the same classes as medications that were included in the Beers Criteria lists (Additional file 1: Table S1).

### Costs of the medications

The costs of the medications were based on the Dispensed Price for Maximum Quantity (DPMQ) for the medications extracted from the PBS [11]. The DPMQ incorporates the “price ex-manufacturer, including all fees, mark-ups and patient contributions” [12]. The medication codes were matched to the DPMQ costs as of the 1st March 2016 PBS i.e. costs at the time of completion of the study. Where a DPMQ for a medication was not available in the 1st March 2016 listings the PBS cost for the nearest available month was used.

The 12 month cost of each participant’s PIMs was calculated and estimated as a proportion of their total medication costs for the 12 months. Costs are reported in Australian dollars (AUD). Where US dollars are reported a March 2016 exchange rate of $1.00 AUD to $0.7814 US was used [13].

### Models of residential care

The INSPIRED study includes some facilities which have adopted a home-like model of residential care, in addition to more standard large-scale facilities. These home-like models of residential care are small houses specifically designed and built to provide person-centred care for residents with dementia, in an environment that looks and feels more like a domestic home. These facilities aim to encourage residents to live in a more independent manner and to contribute to the domestic duties of the units. The facilities which had a home-like model were defined by each having at least five of the following six key components: 1) small size (maximum 15 residents), 2) accessible outdoor areas which residents can use independently, 3) continuity of care staff allocated to units to ensure continuity of care, 4) meals cooked within the units, 5) meals put on table for self-service, 6) residents assist with meal preparation [14]. These descriptive criteria were based on similar models of care described elsewhere [8, 14–16] and were agreed in consultation with an advisory group including consumer representatives from the Alzheimer’s Australia Consumer Dementia Research Network (who were informal caregivers), clinicians, health services researchers, and representatives of long-term care providers.

### Data assessment

Data collected from the participants included PAS-Cog scores which provide an overall measure of cognitive functioning, modified Barthel Index scores which measure activities of daily living, and Neuropsychiatric Inventory (NPI) scores which measure behaviour. Social interaction was defined as visits by family, friends or neighbours at least once a week. Comorbidities were extracted from the medical records of the participants and grouped into one of ten disease categories (excluding dementia) as used by Cohen-Mansfield and colleagues [17]. Data collected regarding the facility included information from a standardised questionnaire based on the work of a previous study [18] and was completed by a member of staff from each facility.

### Statistical analysis

The costs were positively skewed and were therefore log transformed to correct this to a normal distribution. For the total medication costs there were no zero values and therefore multivariate linear models of the transformed costs were used to assess the ratio of mean total medication costs by exposure to a PIM. As the costs of PIMs included many zero values, two-part models were used to assess the ratio of mean total costs of PIMs [19, 20]. First, a logistic regression model was used to predict the probability of any cost of PIMs more than zero in a home-like model of care compared to a standard model of residential care. Second, a log-normal model weighted by the probability of cost more than zero was used to model nonzero cost of PIMS associated with residing in a home-like model of care compared to a standard model of residential care. Models were adjusted for the following potential confounding factors: age, sex, marital status, activities of daily living as measured by the modified Barthel Index, social interactions, number of comorbidities, NPI scores and PAS-Cog scores. The level of statistical significance was set at *p* < 0.05. All analyses were completed using Stata v.14.0 (Stata Corp LP, College Station, TX, USA).

## Results

### Characteristics of the participants

The mean (±SD) age of the participants was 85.5 (±8.5) years old and 74.5% (*n* = 403) were female. Of the total participants, 82.8% (*n* = 448) had some level of cognitive impairment based on their PAS-Cog score and 64.3% (*n* = 348) had a dementia diagnosis.

No medication data were available for eight of the participants; the final sample comprised of 533 participants (98.5%). In this study, 81.4% (*n* = 434) of the participants had been exposed to a PIM during the 12 month period. Of all of the medications dispensed in this period, 15.9% were PIMs. Those who were not exposed to a PIM were more likely to be older (*p* = 0.005), have a diagnosis of dementia (*p* = 0.002) and live in a home-like model of residential care (*p* = 0.02) (Table 1).

**Table 1:** Characteristics of the study participants, by potentially inappropriate medication (PIM) exposure

Characteristic	All participants (*n* = 541)	Participants prescribed at least one PIM (n = 434)	Participants not prescribed a PIM (*n* = 99)	*P*-value
Age, mean (SD)	85.5 (8.5)	84.9 (8.9)	87.9 (6.4)	0.005
Female, n (%)	403 (74.5)	323 (74.4)	77 (77.8)	0.487
Married, n (%)	137 (25.3)	111 (25.7)	24 (24.2)	0.765
Modified Barthel Index, median (IQR)	35.0 (9.0–71.0)	37.0 (10.0–72.0)	25.0 (6.0–62.0)	0.060
Number of comorbidities, mean (SD)	3.7 (1.4)	3.7 (1.4)	3.5 (1.5)	0.348
Neuropsychiatric Inventory, median (IQR)	7.0 (3.0–12.0)	7.0 (3.0–13.0)	6.0 (3.0–11.0)	0.152
Dementia diagnosis, n (%)	348 (64.3)	265 (62.1)	77 (78.6)	0.002
PASCog Score, median (IQR)	15.0 (6.0–21.0)	14.2 (5.0–21.0)	18.0 (9.0–21.0)	0.050
Residing in a home-like model of residential care, n (%)	120 (22.2)	89 (20.5)	31 (31.3)	0.020

Based on the standard list of PIMs from the Beers Criteria for all older adults, not including the additional list of PIMs for older adults with dementia

*Abbreviations: IQR* Inter-quartile range, *PAS-Cog* Psychogeriatric Assessment Scale – Cognitive Impairment Scale

*P* values are from chi-squared or Mann-Whitney tests

### Costs of all medications over 12 months

The mean (±SD) number of different types of medications that participants were exposed to over the 12 month period was 14.5 (±6.5). The mean (±SD) cost for all medications in the 12 month period was AUD$1991.86 (±1538.76) (US$1556.44 (±1202.47)).

### Costs of potentially inappropriate medications

Table 2 shows the proportions of each type of PIM using the list of PIMs for all older adults. The most common PIMs included proton-pump inhibitors prescribed for > 8 weeks (42.2% exposed to a proton-pump inhibitor prescribed for > 8 weeks in the previous 12 months), benzodiazepines (37.9%) and antipsychotics (30.6%). The prevalence of other PIMs were all relatively low (< 10%). The prevalence of antidepressants classified as PIMs was 6.4%, however, the prevalence of any antidepressant was high (52.5%) and these should be used with caution in older adults according to the Beers Criteria.

**Table 2:** Number of participants prescribed potentially inappropriate medications over a 12 month period

PIM	All participants^a^ (*n* = 533), n %	Participants with cognitive impairment and dementia^b^ (*n* = 461), n (%)
Any PIM	434 (81.4)	375 (81.3)
Antidepressants	34 (6.4)	22 (4.8)
Antiemetic	n/a	26 (5.6)
Antihistamines^c^	3 (0.6)	11 (2.4)
Anti-infective	27 (5.1)	24 (5.2)
Antimuscarinics	n/a	13 (2.8)
Antiparkinsonian agents	2 (0.4)	1 (0.2)
Antispasmodics	2 (0.4)	2 (0.4)
Antipsychotics	163 (30.6)	157 (34.1)
Antithrombotics	14 (2.6)	11 (2.4)
Benzodiazepines	202 (37.9)	166 (36.0)
Cardiovascular^d^	40 (7.5)	35 (7.6)
Central alpha blockers	6 (1.1)	5 (1.1)
Endocrine	52 (9.8)	41 (8.9)
Gastrointestinal^e^	51 (9.6)	42 (9.1)
H_2_-receptor antagonists	n/a	12 (2.6)
Pain medications	29 (5.4)	24 (5.2)
Peripheral alpha-1 blockers	8 (1.5)	6 (1.3)
Proton-pump inhibitors	225 (42.2)	183 (39.7)

^a^Includes all study participants with complete medication data and PIMs were based on the standard list of PIMs from the Beers Criteria for all older adults

^b^Participants with cognitive impairment and dementia: sub-group analysis which only includes participants with a PAS-Cog score > 4 or a formal diagnosis of dementia and the PIMs were based on the standard list of PIMs from the Beers Criteria for all older adults and the additional list of PIMs from the Beers Criteria for people with cognitive impairment and dementia. ^c^First generation antihistamines only considered for PIMs for older adults; ^d^digoxin, nifedipine and amiodarone; ^e^metoclopramide

Table 3 shows the costs of the PIMs. The mean (±SD) cost for all PIMs in the 12 month period was AUD$410.89 (±479.45) (US$ 327.07 (±374.64)) per participant. The mean (±SD) proportion of total medication costs that were PIMs was 17.5% (±17.8). When examining the different classes of PIMs the largest mean (±SD) costs in the 12 month period were for proton-pump inhibitors (AUD$139.54 (±163.58) US$109.04 (±127.82)) and antipsychotics (AUD$85.11 (±202.83) US$66.50 (±158.49)). Those who were exposed to a PIM were more likely to have higher total medication costs (β = 0.307, 95% CI 0.235 to 0.379, *p* < 0.001), after adjusting for potential confounding factors (Table 4).

**Table 3:** The estimated costs of medications in a 12 month period amongst participants prescribed a potentially inappropriate medication

All participants who were prescribed at least one PIM^a^ (*n* = 434)	Costs (AUD$), mean (SD)	Proportion of total PIM costs, mean % (SD)
Total PIMs cost	410.89 (479.45)	–
Daily PIMs cost	0.92 (1.26)	–
Cost of PIMs by type of medication		
Antidepressants	8.21 (32.63)	2.1 (9.2)
Antihistamines^c^	0.87 (12.60)	0.4 (5.5)
Anti-infective	8.30 (44.17)	2.1 (10.8)
Antiparkinsonian agents	0.37 (5.99)	0.1 (1.2)
Antispasmodics	0.83 (16.09)	0.2 (2.8)
Antipsychotics	85.11 (202.83)	21.0 (33.6)
Antithrombotics	8.57 (53.79)	1.6 (10.3)
Benzodiazepines	54.78 (93.58)	18.7 (29.1)
Cardiovascular^d^	5.40 (26.16)	4.8 (19.8)
Central alpha blockers	3.45 (29.98)	0.5 (5.9)
Endocrine	77.23 (382.14)	7.4 (22.5)
Gastrointestinal^e^	6.30 (38.54)	3.0 (13.6)
Pain medications	10.23 (54.50)	3.4 (15.2)
Peripheral alpha-1 blockers	1.71 (13.82)	0.4 (3.8)
Proton-pump inhibitors	139.54 (163.58)	34.4 (38.3)
Participants with cognitive impairment or dementia who were prescribed at least one PIM^b^ (*n* = 375)		
Total PIMs cost	416.93 (499.86)	–
Daily PIMs cost	1.14 (1.37)	–
Cost of PIMs by type of medication		
Antidepressants	6.66 (31.34)	1.6 (8.3)
Antiemetic	9.24 (58.76)	1.5 (8.6)
Antihistamines	4.83 (38.06)	1.3 (8.9)
Anti-infective	8.72 (46.04)	2.2 (11.2)
Antimuscarinics	3.15 (22.88)	1.0 (7.0)
Antiparkinsonian agents	0.31 (6.09)	0.03 (0.7)
Antispasmodics	0.96 (17.31)	0.2 (3.1)
Antipsychotics	93.76 (211.87)	22.7 (34.1)
Antithrombotics	6.84 (46.89)	1.3 (8.8)
Benzodiazepines	50.59 (89.44)	17.3 (28.0)
Cardiovascular^d^	5.62 (27.61)	4.9 (20.3)
Central alpha blockers	3.29 (29.33)	0.5 (6.1)
Endocrine	74.39 (394.53)	6.8 (21.8)
Gastrointestinal^e^	6.11 (37.88)	3.0 (13.6)
H_2_-receptor antagonists	4.60 (27.99)	1.6 (10.3)
Pain medications	10.39 (56.89)	3.2 (14.6)
Peripheral alpha-1 blockers	1.42 (12.74)	0.3 (3.6)
Proton-pump inhibitors	126.07 (156.35)	30.5 (36.3)

^a^Includes all study participants with complete medication data exposed to a PIM in the 12 month period; PIMs were based on the Beers Criteria for all older adults

^b^Participants with cognitive impairment and dementia: sub-group analysis includes participants with a PAS-Cog score > 4 or a formal diagnosis of dementia exposed to a PIM in the 12 month period; PIMs were based on the standard list of PIMs from the Beers Criteria for all older adults and the additional list of PIMs from the Beers Criteria for people with cognitive impairment and dementia

All costs are based on Dispensed Price for Maximum Quantity (DPMQ) pricing from the Pharmaceutical Benefits Scheme (PBS). ^c^Only first generation antihistamines are considered for PIMs for all older adults; ^d^digoxin, nifedipine and amiodarone; ^e^metoclopramide

**Table 4:** Associations between exposure to potentially inappropriate medications and total medication costs: log transformed linear regression models

	β (95% CI)	*P*-value
All participants		
Exposed to a PIM, Unadjusted model	0.333 (0.261, 0.406)	<0.001
Exposed to a PIM, Adjusted model^a^	0.307 (0.235, 0.379)	<0.001
Participants with cognitive impairment or dementia		
Exposed to a PIM, Unadjusted model	0.341 (0.264, 0.417)	<0.001
Exposed to a PIM, Adjusted model^a^	0.319 (0.243, 0.395)	<0.001

Reference group is participants not exposed to a PIM

^a^Log transformed linear regression models adjusted for age, sex, marital status, activities of daily living as measured by the modified Barthel Index, social interactions, number of comorbidities, Neuropsychiatric Inventory (NPI) scores and PAS-Cog scores

PIMs were based on the standard list of PIMs from the Beers Criteria for all older adults

Participants with cognitive impairment and dementia: sub-group analysis includes participants with a PAS-Cog score > 4 or a formal diagnosis of dementia exposed to a PIM in the 12 month period; PIMs were based on the standard list of PIMs from the Beers Criteria for all older adults and the additional list of PIMs from the Beers Criteria for people with cognitive impairment and dementia

All costs are based on Dispensed Price for Maximum Quantity (DPMQ) pricing from the Pharmaceutical Benefits Scheme (PBS)

### Potentially inappropriate medications by model of residential care

Four facilities (23.5%) were classified as a home-like model of residential care including 22.2% (*n* = 120) of the participants. These home-like facilities all provided specialised dementia care and 98.3% of the participants in these facilities had a diagnosis of dementia. The first part of the two-part model (logistic regression model) estimates a significant odds ratio of e^(−0.735)^ = 0.48 (*p* = 0.008), indicating that the odds of incurring any costs from PIMs was 52% lower for those living in a home-like model of residential care, after adjusting for potential confounding factors. The second part of the model (log-normal model) indicates residing in a home-like model of care was marginally associated (*p* = 0.064) with lower costs of PIMs over 12 months (mean cost ratio = e^-0.277^ = 75.8%, therefore 24.2% lower costs) after accounting for those with zero costs (Table 5).

**Table 5:** Associations between models of residential care and costs of potentially inappropriate medications: two-part models

	Unadjusted		Adjusted^a^	
	β (95% CI)	*P*-value	β (95% CI)	*P*-value
All participants				
First part: logistic regression model				
Residing in a home-like model of care	−0.569 (−1.054, −0.085)	0.021	−0.735 (−1.283, −0.188)	0.008
Second part: log-normal linear model				
Residing in a home-like model of care	−0.191 (−0.461, 0.078)	0.165	−0.277 (−0.570, 0.016)	0.064
Participants with cognitive impairment or dementia				
First part: logistic regression model				
Residing in a home-like model of care	−0.654 (−1.15, 0.160)	0.010	−0.724 (−1.280, −0.169)	0.011
Second part: log-normal linear model				
Residing in a home-like model of care	−0.194 (−0.457, 0.068)	0.147	−0.284 (−0.567, 0.002)	0.051

Reference group is participants residing in a standard Australian model of care

^a^Two-part-models adjusted for age, sex, marital status, activities of daily living as measured by the modified Barthel Index, social interactions, number of comorbidities, Neuropsychiatric Inventory (NPI) scores and PAS-Cog scores

PIMs were based on the standard list of PIMs from the Beers Criteria for all older adults

Participants with cognitive impairment and dementia: sub-group analysis which only includes participants with a PAS-Cog score > 4 or a formal diagnosis of dementia exposed to a PIM in the 12 month period; PIMs were based on the standard list of PIMs from the Beers Criteria for all older adults and the additional list of PIMs from the Beers Criteria for people with cognitive impairment and dementia

All costs are based on Dispensed Price for Maximum Quantity (DPMQ) pricing from the Pharmaceutical Benefits Scheme (PBS)

### Costs of potentially inappropriate medications for dementia and cognitive impairment: Subgroup analysis

In total, 86.5% (*n* = 461) of the participants had dementia or cognitive impairment and were included in this analysis. Of these participants, 81.3% (*n* = 375) had been exposed to a PIM that is not recommended for older adults and/or contraindicated in dementia or cognitive impairment. The mean (±SD) cost for these PIMs in the 12 month period was AUD$416.93 (±499.86). The mean (±SD) proportion of total medication costs that were PIMs for those with cognitive impairment and dementia was 18.3% (±18.3). When examining the different classes of PIMs the largest mean (±SD) costs in the 12 month period were for proton-pump inhibitors prescribed for > 8 weeks (AUD$126.07 ± 156.35) and antipsychotics (AUD$93.76 ± 211.87). The logistic regression model estimates a significant odds ratio of e^(−0.724)^ = 0.48 (*p* = 0.011), indicating that the odds of incurring any costs from PIMs was 52% lower for those living in a home-like model of residential care, after adjusting for potential confounding factors. The log-normal model indicates residing in a homelike model of care was marginally associated (*p* = 0.051) with the lower costs of PIMs over 12 months (mean cost ratio = e^-0.284^ = 75.3%, indicating 24.7% lower costs) after excluding those with zero costs.

## Discussion

This is the first study that has examined the 12 month costs of PIMs in older adults in residential care with a high prevalence of cognitive impairment and dementia using the updated Beers Criteria, to the author’s knowledge. This analysis has demonstrated that over 80% of participants had been exposed to a PIM over a 12 month period and approximately 16% of all medications dispensed in this 12 month period were PIMs. Those exposed to a PIM were more likely to have higher total medication costs. The highest exposures to PIMs were for proton-pump inhibitors prescribed for more than 8 weeks, benzodiazepines and antipsychotics. Furthermore, those who resided in a home-like model of residential care were less likely to incur any costs due to PIMs.

Few previous studies have provided period prevalence estimates for PIMs over 12 months in residential aged care settings. Previous studies have shown the prevalence of PIMs taken at a single time point (point prevalence) by older adults in residential care settings to be approximately 50% [7, 21]. However, this estimate has varied in different populations; in a Brazilian study this was reported to be as high as 82.6%, with 32% of all medications being PIMs [22]. The fact that the period prevalence of PIMs in this study over 12 month period was high is unsurprising. The population examined in this study had a high prevalence of cognitive impairment and dementia; all resided in an aged care facility and the mean age was over 85 years. It is therefore expected that exposure to PIMs would be higher than in studies of community-dwelling older adults or relatively younger age groups. For instance, PIM exposure has been shown to be higher in women aged 85 years and over [23]. The current study adds to the body of evidence by using the 2015 updated Beers Criteria and examining a population of long-term aged care residents with a high prevalence of cognitive impairment and dementia.

In this study the highest exposure to PIMs were due to proton-pump inhibitors, benzodiazepines and antipsychotics. This profile of use is similar to previous studies which have found proton-pump inhibitors to be the largest cause of PIM exposure for older people [24]. Proton-pump inhibitor use in older adults has been associated with an increased risk of bone loss and fractures, *Clostridium difficile* infection, community-acquired pneumonia, and vitamin and mineral deficiencies [25]. There was also high-exposure to psychotropic PIMs (benzodiazepines and antipsychotics), which are frequently associated with adverse effects in older adults, including falls, hospitalization, cardiovascular complications, adverse mental state changes and mortality in older people in residential care [26]. The use of antipsychotics for some of the participants in the current study may be appropriate, given the high prevalence of dementia in this study. However, guidelines recommend use of antipsychotics only in rare cases for those with extreme behavioural and psychological symptoms of dementia (BPSD) [27, 28], and therefore the use of antipsychotics in 30% of the study population at some stage during the previous 12 months indicates that a significant proportion of use is likely to be inappropriate. Furthermore, although we found the prevalence of antidepressants classified as PIMs to be quite low, we found the prevalence of any antidepressant use was high (over 50%). Some of the use of these additional antidepressants (SSRIs and SNRIs) may also be inappropriate in some cases as these medications are recommended to be used with caution in older adults and SSRIs should be avoided in those with a history of falls or fractures according to the Beers Criteria and also antidepressants may not be effective for depression associated with dementia [27].

Deprescribing is defined as the process of withdrawal of an inappropriate medication, supervised by a healthcare professional with the goal of managing polypharmacy and improving outcomes [29]. The high exposure to PIMs in our study indicates that older adults in residential care could be an appropriate target group for deprescribing. This may not only benefit the residents, but may also lead to cost saving. In addition to the direct costs of PIMs there are additional indirect costs of managing associated adverse drug events. Deprescribing of PIMs may reduce both direct and indirect costs associated with their use. However, substitution with alternatives, such as non-pharmacological interventions, carries its own costs. Future studies should investigate the cost-effectiveness of deprescribing PIMs in residential care. Several randomised controlled trials have found positive effects of interventions in residential care facilities (e.g., educational interventions for clinical staff, medication reviews) in reducing the use of inappropriate medications; however, the effects on clinical outcomes remain unclear [30–36].

In Australia, around AUD$9 billion is spent annually (2011–2012 figure) by the government on residential aged care services and in the same year there were 187,941 residential aged care places [37]. Furthermore, medications constitute a high proportion of all direct health costs in residential aged care [38]. Based on the numbers of people exposed to a PIM, the average amount spent on PIMs per participant in this study, and the feasibility of stopping PIMS in practice [39], reducing exposure to half of these PIMs could result in an annual direct saving in medication costs of approximately AUD$38 million in Australia.

Participants who were living in a home-like model of residential care had a lower risk of incurring any costs due to PIMs over the one year period compared to those living in standard models of care. The prevalence and costs of PIMs in different models of residential care have not been previously explored. Residential care facilities which offer a home-like model of care have been shown to favourably impact quality of life, behavioural symptoms and emotional well-being of the residents [8, 10, 40, 41]. Reasons for lower exposure to PIMs in these settings cannot be determined in the current study, but a possible improvement in quality of life seen in the residents may lead to fewer behavioural symptoms and a lower proportion of PIM use in these settings. However, there was no difference in NPI scores between those who had and had not been exposed to a PIM. Also, staff in alternative models of residential care may be able to accommodate and manage changed behaviours more effectively using alternative approaches than is possible in standard models of care. Further research is needed to clarify the effectiveness of home-like care models on different outcomes for residents and staff [10]. Those that had not been exposed to a PIM were more likely to have a diagnosis of dementia and this may be due to a higher proportion of people living with dementia residing in a home-like model of care, but the definitive reason for this association could not be determined in this study.

### Strengths and limitations

This study collected comprehensive data on an older population living in residential care, including a high prevalence of people with cognitive impairment and dementia. The dispensing data were collected from electronic records held by the individual pharmacies used by the facilities and they are inclusive of all of the medications dispensed to the individuals over the study period.

We were unable to include PIMs based on diagnosis (apart from cognitive impairment and dementia); therefore only medications which are PIMs for all older adults were included so this may be an underestimate of the actual costs of PIMs in this population. There is also a difference in formularies between Australia and the US where the Beers Criteria were developed as some medications are not available in Australia and some medications were added to adapt the Beers Criteria to an Australian setting (4% of PIMs were additional medications added). Polypharmacy has been identified as a risk factor for exposure to PIMs [7]. However, we could not directly examine the prevalence of polypharmacy in this study as we examined 12 month period prevalence of medications rather than point prevalence.

Facilities which have adopted a home-like model of care as described in this study were all located in one state in Australia and these facilities are specifically designed for people with dementia. However, we have adjusted for many potential confounding factors including PAS-Cog scores and NPI scores between the models of care.

## Conclusions

Exposure to PIMs is very common amongst older adults with a high prevalence of cognitive impairment and dementia permanently residing in aged care facilities. The financial cost of PIMs is high and has the potential to be lowered by reducing the prevalence of PIMs in this setting. Facilities which had adopted a home-like model of residential care were less likely to incur costs due to PIMs. Further research should investigate whether deprescribing of PIMs in older people in residential facilities could lead to cost savings and improvements in outcomes such as mortality, hospitalization and quality of life.

## Additional file

Additional file 1: Table S1. Complete list of medications considered potentially inappropriate, according to the Beers Criteria, and adapted for an Australian setting. (DOCX 31 kb)

## Abbreviations

AUD: Australian dollars
BPSD: Behavioural and psychological symptoms of dementia
CI: Confidence interval
DPMQ: Dispensed Price for Maximum Quantity
INSPIRED: Investigating Services Provided in the Residential Environment for Dementia
NPI: Neuropsychiatric Inventory
PAS-Cog: Psychogeriatric Assessment Scale-Cognitive Impairment Scale
PBS: Pharmaceutical Benefits Scheme
PIM: Potentially inappropriate medication
SD: Standard deviation
