# Supplementary material for: Costs of potentially inappropriate medication use in residential aged care facilities
Source: BMC Geriatr. 2018 Jan 11;18:9. doi: 10.1186/s12877-018-0704-8 (PMC5765623; doi:10.1186/s12877-018-0704-8)
Supplement: Additional file 1: Table S1. — Complete list of medications considered potentially inappropriate, according to the Beers Criteria, and adapted for an Australian setting. (DOCX 31 kb) [file 12877_2018_704_MOESM1_ESM.docx]

**Additional file 1: Table S1. Complete list of medications considered potentially inappropriate, according to the Beers Criteria, and adapted for an Australian setting.**

| **PIMs for all older adults** | **Additional PIMs for adults with cognitive impairment or dementia** |
| --- | --- |
| *First generation antihistamines* | *Antihistamines* |
| Brompheniramine | Carbinoxamine |
| Chlorpheniramine | Cetirizine* |
| Cyproheptadine* | Clemastine |
| Dexchlorpheniramine | Cyclizine |
| Dimenhydramine (oral) | Desloratadine* |
| Doxylamine | Dexbrompheniramine |
| Promethazine* | Fexofenadine* |
| Triprolidine | Hydroxyzine |
|  | Loratadine* |
| *Antiparkinsonian agents* | Pheniramine |
| Benzatropine* | Trimeprazine |
| Biperiden* |  |
| Trihexyphenidyl* | *Antimuscarinics (urinary incontinence)* |
|  | Darifenacin |
| *Antispasmodics* | Fesoterodine |
| Atropine (excludes opthalmic) | Flavoxate |
| Belladonna alkaloids | Oxybutynin* |
| Hyoscyamine | Propantheline |
| Propantheline* | Solifenacin* |
| Scopolamine* | Tolterodine |
|  | Trospium |
| *Antithrombotics* |  |
| Dipyridamole, oral short-acting* | *Antiemetic* |
| Ticlopidine | Domperidone* |
|  | Droperidol |
| *Anti-infective* | Prochlorperazine |
| Nitrofurantoin* | Promethazine* |
|  |  |
| *Peripheral alpha-1 blockers* | *H2-receptor antagonists* |
| Prazosin* | Cimetidine |
| Terazosin | Famotidine |
|  | Nizatidine* |
| *Central alpha blockers* | Ranitidine* |
| Clonidine |  |
| Disopyramide |  |
| Methyldopa* |  |
| Moxonidine* |  |
|  |  |
| *Other cardiovascular medications* |  |
| Amiodarone* |  |
| Digoxin* |  |
| Nifedipine, immediate release* |  |
|  |  |
| *Antidepressants, alone or in combination* |  |
| Amitriptyline* |  |
| Clomipramine* |  |
| Doxepin (>6mg/day)* |  |
| Imipramine |  |
| Nortriptyline* |  |
| Paroxetine* |  |
|  |  |
| *Antipsychotics (first and second generation)*  Amisulpride  Aripiprazole*  Asenapine  Chlorpromazine*  Clozapine  Droperidol  Flupentixol*  Fluphenazine  Haloperidol*  Lurasidone  Olanzapine*  Paliperidone  Periciazine*  Quetiapine*  Risperidone*  Trifluoperazine  Ziprasidone  Zuclopenthixol  *Barbituates*  Phenobarbital  Primidone  *Benzodiazepines (short and immediate acting)*  Alprazolam*  Bromazepam*  Clobazam  Flunitrazepam*  Lorazepam*  Midazolam*  Nitrazepam*  Oxazepam*  Temazepam*  Triazolam  *Benzodiazepines (immediate acting)*  Clonazepam*  Diazepam*  *Non benzodiazepine benzodiazepine receptor agonist hypnotics*  Zolpidem  *Endocrine*  Testosterone*  Estrogens with or without progestins (not vaginal creams)  Progestogens and estrogens, fixed combinations (not vaginal creams)  Glibenclamide  Gliclazide*  Glimepiride*  Glipizide*  Growth hormone  Insulin, sliding scale*  Megestrol  *Gastrointestinal*  Metoclopramide*  Mineral oil, given orally  *Proton-pump inhibitors >8 weeks*  Esomeprazole*  Lansoprazole*  Omeprazole*  Pantoprazole*  Rabeprazole*  *Pain medications*  Aspirin >325mg/day  Celecoxib*  Etoricoxib  Ibuprofen  Indomethacin  Ketoprofen  Ketorolac, includes parenteral  Mefenamic acid  Meloxicam*  Naproxen  Parecoxib  Pentazocine  Pethidine  Piroxicam  Sulindac  *Skeletal muscle relaxants*  Orphenadrine  *Genitourinary*  Desmopressin |  |

Medications were added to the list including medications that are in the same classes as medications that were listed in the Beers Criteria and are available in Australia. Medications that are no longer available in Australia are not listed here.

*Prescribed to participants of the INSPIRED study.
